# Accurate and intelligent diagnosis of pediatric pneumonia using X-ray images and blood testing data

**DOI:** 10.3389/fbioe.2023.1058888

**Published:** 2023-05-17

**Authors:** Dan Yao, Zhenghua Xu, Yi Lin, Yuefu Zhan

**Affiliations:** ^1^ State Key Laboratory of Reliability and Intelligence of Electrical Equipment, School of Health Sciences and Biomedical Engineering, Hebei University of Technology, Tianjin, China; ^2^ Department of Radiology, Hainan Women and Children’s Medical Center, Haikou, China

**Keywords:** pediatric pneumonia diagnosis, X-ray images, blood testing, multimodal feature fusion, two-stage deep model

## Abstract

Computer-aided diagnosis (CAD) methods such as the X-rays-based method is one of the cheapest and safe alternative options to diagnose the disease compared to other alternatives such as Computed Tomography (CT) scan, and so on. However, according to our experiments on X-ray public datasets and real clinical datasets, we found that there are two challenges in the current classification of pneumonia: existing public datasets have been preprocessed too well, making the accuracy of the results relatively high; existing models have weak ability to extract features from the clinical pneumonia X-ray dataset. To solve the dataset problems, we collected a new dataset of pediatric pneumonia with labels obtained through a comprehensive pathogen-radiology-clinical diagnostic screening. Then, to accurately capture the important features in imbalanced data, based on the new dataset, we proposed for the first time a two-stage training multimodal pneumonia classification method combining X-ray images and blood testing data, which improves the image feature extraction ability through a global-local attention module and mitigate the influence of class imbalance data on the results through the two-stage training strategy. In experiments, the performance of our proposed model is the best on new clinical data and outperforms the diagnostic accuracy of four experienced radiologists. Through further research on the performance of various blood testing indicators in the model, we analyzed the conclusions that are helpful for radiologists to diagnose.

## 1 Introduction

Pneumonia refers to severe inflammation caused by infections inside the lungs, which are crucial organs of the respiratory system. In particular, among diseases of infants and young children, pneumonia has a high fatality rate. Among children under 5 years of age, pneumonia accounts for 14% of all childhood deaths from the disease (Troeger et al., 2018). Due to the growth stage of children, CT and other radioactive imaging methods should be avoided. However, in clinical practice, we often need to accurately diagnose the type of disease to be able to use targeted drugs to avoid the impact of antibiotic abuse and drug side effects on children’s growth and development. Therefore, accurate classification of pneumonia in children based on X-ray low-radiation imaging modalities is a challenge.

**FIGURE 1:**
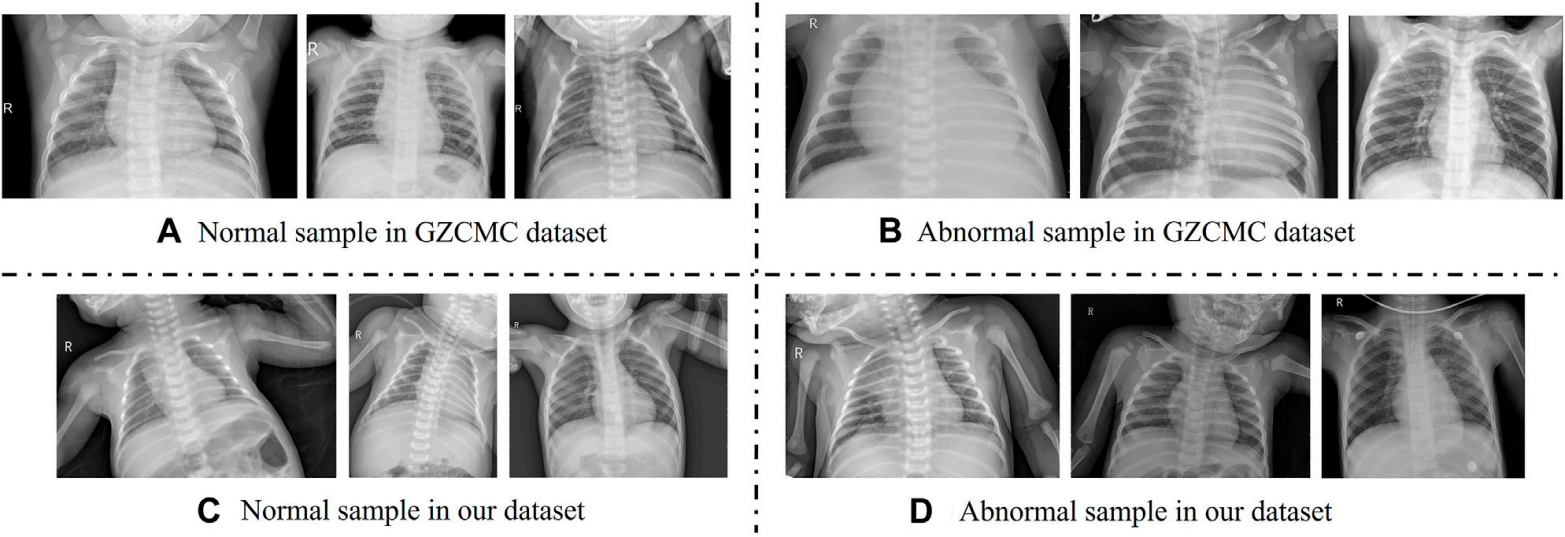
Comparison with adolescent and infant X-ray samples from the Guangzhou Women and Children’s Medical Center (GZCMC) Dataset. **(A,B)** Represent samples the GZCMC dataset. **(C,D)** Represent samples from our dataset.

With the development of deep learning technology, automatic diagnosis and treatment technology based on deep learning has been widely applied in the classification of children’s pneumonia. Most of the studies on the classification of pneumonia focus on the two-class problem of diagnosing whether a patient has pneumonia (Baltruschat et al., 2019; Siddiqi, 2019; Liz et al., 2021), including diagnosing whether a patient has epidemic pneumonia, COVID-19, etc., and few studies focus on three-class problems (Rajpurkar et al., 2017). Then, according to the results of these researches, the classic Resnet can get a performance along 86% on the dataset of Guangzhou Women and Children’s Medical Centre in China (GZCMC). However, when we put the same model into the actual clinical data of a medical institution similar to Guangzhou Women and Children’s Medical Centre for a retrospective study, the performance of the model dropped very sharply. Specifically, we selected all X-ray images of infants and young children aged 0–14 who were cleared of pneumonia types through pathogen-radiology-clinical diagnostic screening in the Women and Children’s Medical Centre of Hainan Province from 2016 to 2021 as samples to test the effect of the Resnet model. We found that the performance of the model dropped from 80 to 50. According to related work and our experimental results, we identify two challenges in pneumonia classification research: 1) The existing public datasets have undergone too perfect preprocessing and possible data selection. For example, in Figure 1, the first row of overall image shooting locations is relatively regular, concentrated on the lungs, and the image abnormalities are more obvious. However, in actual clinical practice, we often encounter the second row, where infants and young children have different postures and shooting areas. There is more irrelevant information than diagnosis. Therefore, we believe that a model that achieves good results on such carefully selected and preconditioned models is not well suited for clinical use. 2) The existing model has poor feature extraction ability on real clinical pneumonia X-ray images in the imbalanced dataset. Due to the uneven quality of actual clinical data, the classification models of existing related work have limited feature extraction capabilities in unbalanced datasets, resulting in unsatisfactory performance of pneumonia classification models. Therefore, to accurately, automatically, and intelligently classify children’s pneumonia: in fact, we first need a data set that is more representative of the actual clinical situation, and second, we need a deep learning method that has achieved good results on this clinical dataset.

Consequently, in this work, we collected X-ray images and blood testing data of all infants and young children aged 0–14 years old who had confirmed the type of pneumonia through pathogen-radiology-clinical diagnosis screening in the Women and Children’s Medical Centre of Hainan Province from 2016 to 2021. Compared to the previous single-modality pneumonia dataset consisting only of X-ray images, we propose, for the first time, a multi-modality pneumonia dataset that includes both X-ray images and blood test data. We collect all data regardless of image quality and effect. Furthermore, based on the above data sets, we found that the existing resnet, denseness, and other baseline effects are not satisfactory. We think that the quality of data in actual clinical practice is often uneven, and we cannot select data in clinical practice, so its characteristics are not obvious and stable enough. Therefore, for the X-ray pneumonia images of children with uneven quality levels, we propose a more effective and accurate automatic classification method for children’s pneumonia in real clinical situations–attention multimodal pneumonia diagnosis network (AMPNet), which combines X-ray Pneumonia imaging and blood testing information (Mardian et al., 2021) for accurate pneumonia classification. Specifically, our model includes three parts: image feature extraction based on local-global attention, blood detection feature extraction, and modality fusion. In the image feature extraction part, we propose a global attention module and a local feature extraction module to improve the model’s extraction of local subtle features and attention to global important features to ensure that important image features can be captured. In the feature extraction part of blood detection, we use one-dimensional convolution to extract features. In the modality fusion section, we fuse the features of the two modalities and perform pneumonia classification. In addition, our two-stage training strategy can significantly reduce the impact of class-imbalanced data on model performance.

The contributions of this paper are as follows.

• To overcome the existing dataset problem in the current pneumonia classification research, we constructed a multi-modal dataset that can represent the images and blood testing data of children with actual clinical pneumonia. This dataset collected X-ray images of all infants and young children aged 0–14 years old who had confirmed the type of pneumonia through pathogen-radiology-clinical diagnosis screening in the Women and Children’s Medical Centre of Hainan Province from 2016 to 2021. This dataset will be desensitized (i.e., removing sensitive information) and released online after the acceptance of this paper.
• To address the poor feature extraction problem of the existing deep classification models, we further proposed a satisfactory method for the above-mentioned actual clinical X-ray children pneumonia impact classification task. This method includes image feature extraction based on local-global attention, blood detection feature extraction, and modality fusion. The two-stage training strategy is implemented to achieve better performance on clinical data.
• Extensive experimental studies have been conducted on GZCMC and our proposed datasets and the results show the following: 1) Using the proposed method achieves the best results among existing models on real pneumonia clinical data. 2) Ablation experiments demonstrate that the modality fusion method outperforms any single modality method, and the three proposed improvements are effective and essential for the model. 3) The results of our model are much higher than the performance of four experienced radiologists, which has good prospects for clinical practical application.

Overall, we present the dataset required for the experiments and our proposed two-stage multimodal diagnostic model in Section 3. In Section 4, we present all the experimental results we obtained and give an explanation of the experimental results. In Section 5, we analyze the experimental results in more depth and give possible future research directions.

## 2 Related work

### 2.1 X-ray classification of pneumonia

In recent years, various CNN-based methods have been proposed to address the problem of diagnosing pneumonia from chest X-ray images (Bardou et al., 2018; Chouhan et al., 2020; Guan and Huang, 2020). These studies are roughly divided into three types: 1) Optimizing classical deep learning model; 2) Transfer learning pre-training CNN architecture; 3) Integrated models of multiple CNN architectures. In (Siddiqi, 2019; Stephen et al., 2019), the authors proposed specialized CNN architectures for the identification of pneumonia from chest X-ray images, which provided promising classification performance. In order to further improve the feature extraction ability of the model, (Sitaula and Aryal, 2021), proposed a new Bag of Deep Visual Words (BoDVW) method over deep features based on VGG16, which can improve the model’s ability to retain the semantics of each feature map. In addition, (Sitaula and Hossain, 2021), utilized the attention mechanism to capture the spatial relationship between ROIs in X-ray images and improve the performance of pneumonia classification. However, the architectures did not address time complexity or generalization issues. Recently, various studies (Kermany et al., 2018; Baltruschat et al., 2019) have shown that utilizing transfer learning methods provides us with high classification performance. We can leverage different pre-trained CNN architectures without large labeled datasets. While these methods are the most promising, we must consider the problems that arise with the use of transfer learning. These problems often appear when we choose the most appropriate fine-tuning layer combination (Vrbancic and Podgorelec, 2020) and train complex CNN architectures on small datasets. We also call them regularization problems. In addition, the integrated approach also showed excellent results in the diagnosis of pneumonia chest radiographs. (Chouhan et al., 2020). trained AlexNet, DenseNet121, Inception v3, GoogleNet 50, and ResNet-18 individually on the training subset of the GWCMC dataset. Subsequently, they developed an ensemble model with majority voting, achieving 96.4% accuracy (Liz et al., 2021). proposed an ensemble deep learning model based on the CNN model and trained it on the dataset they collected. They also verified the model on the GWCMC dataset, and the AUC can reach 0.92. However, models are affected by data generalization, dataset size, and time complexity. To this end, (Vrbančič and Podgorelec, 2022), developed an ensemble method based on stochastic gradient descent with thermodynamic restart (SGDRE). And they got a two-class accuracy of 96.26% in the GWCMC dataset. All the above studies are based on the CNN structure and have not improved the internal structure of the model, resulting in a limited performance of the model to extract features from images. Although our work is also based on CNN, we found the problem of insufficient feature extraction ability of the model in the existing work and proposed a local and global attention module that can extract more refined image features with the local attention module and find “interesting” feature maps with global attention module. Thus it can improve the classification performance of the model.

### 2.2 Medical image and testing data diagnosis

It is common to study pneumonia classification tasks through image feature extraction. However, these studies only use a single form of information to diagnose pneumonia, ignoring other more easily obtained clinical test result information. Therefore, it is necessary to study the model through multiple modalities in different dimensions and improve the diagnostic performance. At present, there are many multimodal diagnostic methods using clinical detection data (Liu et al., 2019). proposed an extended learning system to detect the construction of medical text data covering various physiological parameters of the human body. Then they used the medical literature data from deep learning networks to predict disease conditions (Ali et al., 2020). combined information from sensor data and electronic medical records to build a smart medical system for predicting cardiac disease using deep learning models. To classify liver tumors, (Zhen et al., 2020), used MR images and multimodal clinical data including text and laboratory test results to build a deep-learning model. Similarly, like (Zhen et al., 2020), we also use the feature extraction module to extract X-ray image features and blood testing features and then fuse the two features for classification tasks. Different from these works, we focus on X-ray images and blood detection data in imbalanced data. Therefore, we propose a two-stage training method: the first stage screens patients for disease, and the second stage identifies the disease the patient suffers from. This method has also been proven to alleviate the multimodal data overfitting problem caused by data imbalance. However, these studies tend to be dichotomous diagnoses of disease. We know that the accuracy of deep learning models decreases significantly with increasing classification types (Rajpurkar et al., 2017). Especially in the diagnosis of pneumonia, the distinction between bacterial pneumonia and viral pneumonia is also a great challenge to doctors in medical diagnosis. Therefore, multi-modal medical diagnostic models for multi-classification are worth studying.

## 3 Materials and methods

### 3.1 Dataset introduction and clinical data collection

Two datasets are used for the study: a public dataset from the Guangzhou Women and Children’s Medical Centre in China (GZCMC) 1 (Kermany et al., 2018) and a dataset on pediatric pneumonia that we collected at the Hainan Women and Children’s Medical Center (Women and Children’s Healthcare Center of Hainan Province, Hainan Children’s Hospital, Children’s Hospital of Fudan University at Hainan, Hainan Obstetrics and Gynecology Hospital). We describe the two datasets in more detail below.

### 3.1.1 Guangzhou women’s and children’s medical center (GZCMC) dataset

The Guangzhou Women and Children Medical Centre dataset (GZCMC) contains 5856 frontal lobe pediatric chest radiographs of pediatric patients aged between 1 and 5 years. The dataset is divided into a training set and a test set, which are currently publicly available. Through manual data selection and expert proofreading, the training set contains 5232 pleural, 3883 pneumonia images (2538 bacterial and 1345 viral), and 1349 realistic normal images. The test set consisted of 624 images, of which 390 are pneumonia images (242 bacterial and 148 viral) and 234 are normal chest images. Two doctors labeled all the images and a third doctor validated all the labels of the test data set. It is unclear what (if any) additional clinical criteria are used to determine these labels. The Guangzhou Women’s and Children’s Medical Centre dataset meets the objectives of our task and serves as a comparison dataset to the clinical datasets we collected. In addition, the GZCMC dataset can be used as a pre-training dataset to fine-tune the basis of the clinical dataset, improving the model’s ability to capture pneumonia-related features and further improving the accuracy of pneumonia diagnosis.

### 3.1.2 Hainan women’s and children’s medical centre dataset

Our clinical pediatric pneumonia data collected clinical pneumonia X-ray images and blood testing results from children aged 0–14 years from July 2016 to September 2021 through the Women and Children’s Medical Centre of Hainan Province and identified the causative pathogens by pathogenic examination to determine the type of pneumonia. Specifically, the dataset contains 2301 normal images, 575 images of bacterial pneumonia, and 224 images of viral pneumonia, of which only bacterial and viral pneumonia include blood test values. X-ray images range in width from 512 to 3408 and in height from 512 to 3032. The dataset contains more invasive information (e.g., the hand used by the doctor to immobilize the child), and a wide age range of children. All of these are more consistent with the characteristics of most truly collected chest X-ray images of children. Blood testing contains clinically obtained indicators such as leucocytes, neutrophils, C-reactive protein, and calcitonin, which are useful in identifying the type of pneumonia agent. The dataset contains a large amount of authentic clinical data while ensuring patient privacy. The study was approved by the Institutional Review Board of the Hainan Women’s and Children’s Medical Centre and all written informed consent was waived.

### 3.2 Data preprocessing

Our dataset images are stored as Digital Imaging and Communications in Medicine (DICOM) files, and DICOM files are converted to images using RadiAnt DICOM. The large differences in the posture and size of the lung area in the imaged children make the analysis of children’s lung X-ray images difficult. To overcome this problem, we used a target detection algorithm (FasterRCNN) to crop out the lung area and unified it into a 512*512 pixel image, with the data normalized. The blood indicators are in excel file format, but only contain indicator data for those suffering from pneumonia. To meet the needs of the model we randomly generated blood indicator values for healthy children based on normal blood indicator thresholds. Our dataset is divided into three parts: 70% for training, 10% for validation and 20% for testing, i.e. 2170 images for training, 310 images for validation and 620 images for testing.

### 3.3 Physician’s clinical blind review standard

To compare the validity of the AMPNet model, we compared the diagnoses of four radiologists in an experiment. The clinical readings in the experiment are performed independently by four radiologists, all of whom had completed the national residency training, two of whom are radiology residents (with 3 and 4 years of experience in interpreting chest images, respectively), and two of whom are attending radiologists (with 7 and 8 years of experience in interpreting chest images, respectively). They are unaware of the clinical information and past imaging findings.

### 3.4 Deep learning multimodal diagnostic model

To expand the possibility of multimodal model research for pneumonia diagnosis and solve the current problem of low accuracy of multi-classification of pneumonia, we propose a two-stage attention multimodal pneumonia diagnosis model (AMPNet). First, we proposed the image feature extraction module and the blood feature extraction module to extract the features of the two modalities and perform feature fusion in the fully connected layer. Second, to improve the ability of the model to capture local features and deep global features for pneumonia images, we proposed a local-global hybrid attention module. Finally, we proposed a two-stage training strategy to alleviate imbalanced datasets. The input of AMPNet combines X-ray images and blood texting data. The structure of the AMPNet model is shown in Figure 3 Specifically, the AMPNet is mainly composed of three parts: an attention-based image feature extraction module, a blood testing feature extraction module, and a modal fusion module.

#### 3.4.1 The attention multimodal pneumonia diagnosis network

##### 3.4.1.1 Attention-based image feature extraction module

**FIGURE 2:**
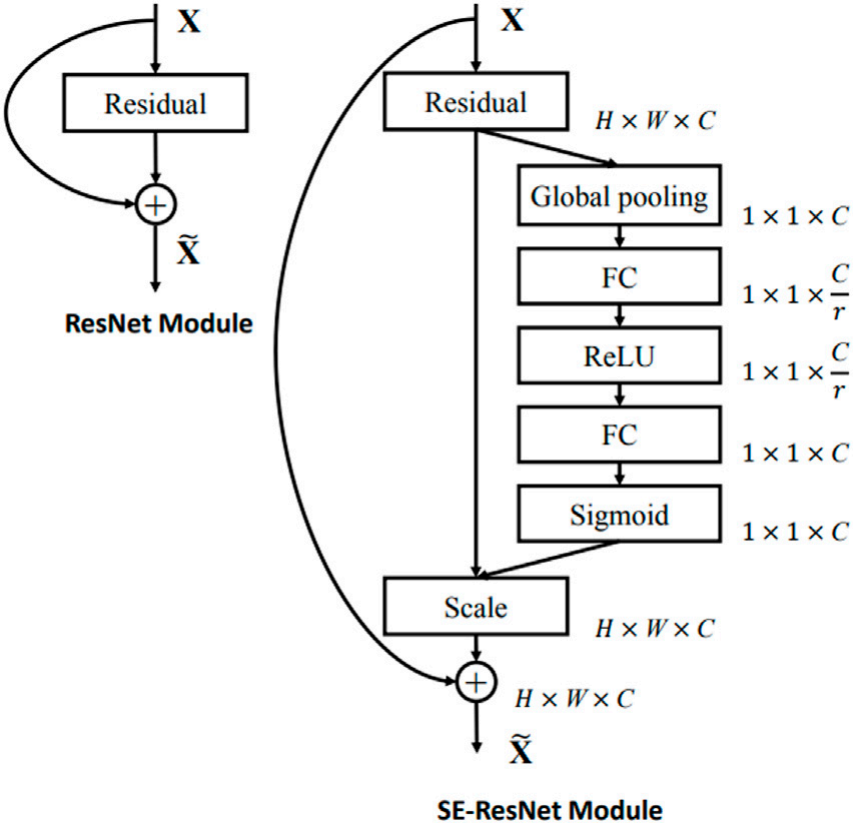
The schema of the original Residual module (left) and the SE-ResNet module (right).

**FIGURE 3:**
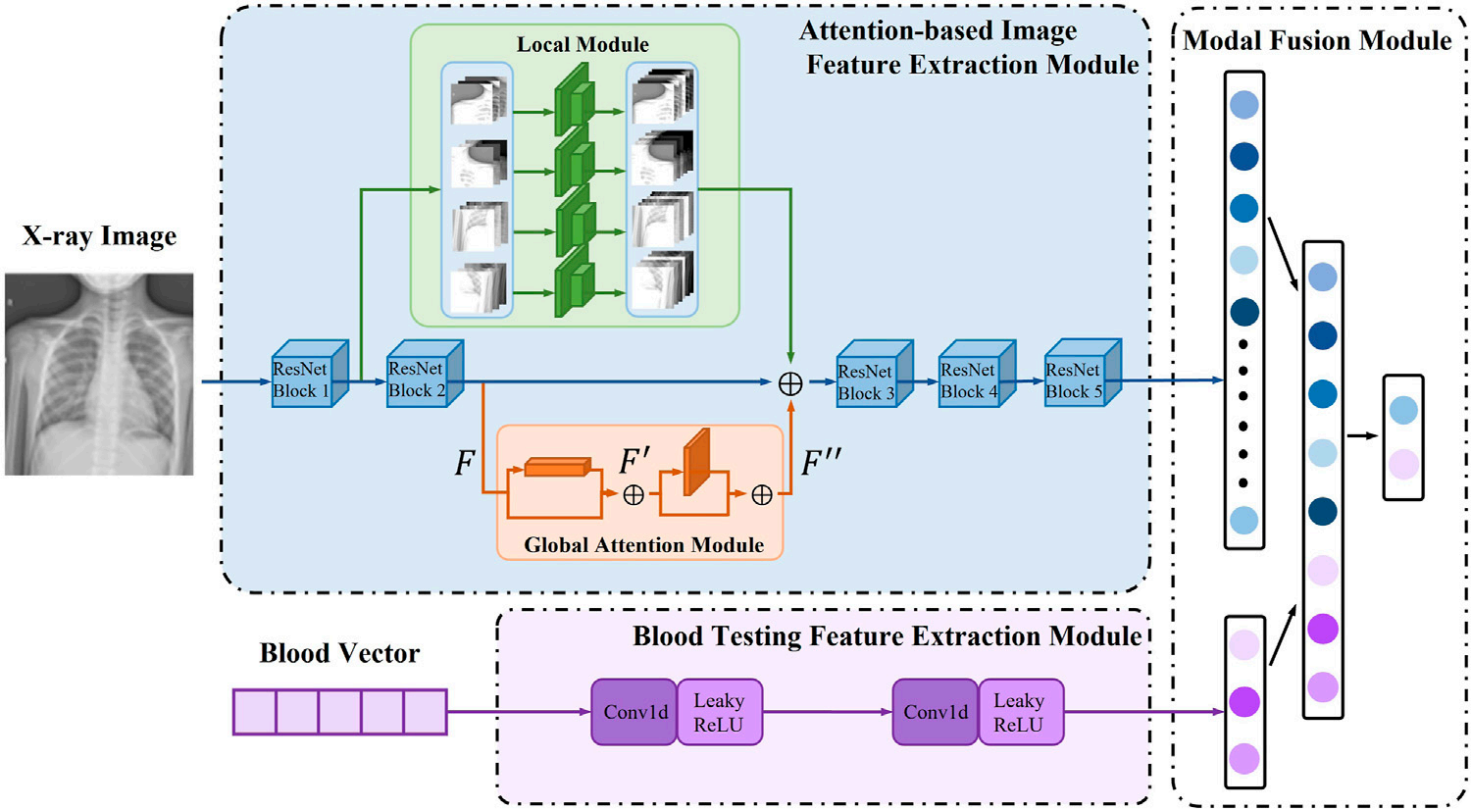
MPNet model structure.

The attention-based image feature extraction module uses seresnet50 as the backbone network, adding a local information extraction module and a global attention module. Seresnet50 is the champion model of the Image Classification task in the ImageNet 2017 competition. This is a fusion model of the resnet50 network (He et al., 2016) and the squeeze-and-excitation network (Hu et al., 2018) proposed by Hu Jie et al. As shown in Figure 2, the left is the residual module (ResNet Block) in the resnet50 network, and the right is the squeeze-and-excitation residual module (SE-ResNet Block) in the seresnet50 network. To capture features that are more interesting for classification models in the global region, we develop a new attention mechanism called global-local hybrid attention, which uses global channel and spatial attention modules to capture features that are important in both channel and spatial dimensions for global deep features, while also extracting local deep features by gridding shallow features. As shown in the Figure 3, we insert the global channel and spatial attention module between the second residual block and the third residual block of the backbone network of seresnet50 and add the local feature extraction module after the first residual block of seresnet50. For the global channel and spatial attention module, we extract the feature map *F* output by the second residual module through the two dimensions of channel and space, and obtains the attention feature *F*″.

$$F^{\prime }=M_{c}\left(F\right)⊗F,F^{\prime \prime }=M_{s}\left(F^{\prime }\right)⊗F^{\prime },$$ (1)

Where *F* ∈ *R* ^*C*×*H*×*W*^ is taken as input, *M*_*c*_ ∈ *R* ^*C*×1×1^ is a 1D channel attention map, and *M*_*s*_ ∈ *R* ^1×*H*×*W*^ is a 2D spatial attention map, as shown in the global attention module in Figure 3 Where ⊗ means bitwise multiplication. When multiplying bitwise, the attention values are broadcast accordingly: channel attention values are broadcast along the spatial dimension and *vice versa*. *F*″ is the output of the final refinement.

The channel feature extraction is as follows:

$$M_{c}\left(F\right)=\sigma \left(MLP\left(AvgPool\left(F\right)\right)\right)+MLP\left(MaxPool\left(F\right)\right),$$ (2)

The spatial information of the feature map *F* is aggregated through the average pooling (AvgPooL) and maximum pooling (MaxPooL) operations to generate two different spatial context descriptions, which represent the average pooled features and the maximum pooled features respectively. Then, these two descriptions are fed forward into a multi-layer perceptron (MLP) network shared by both to generate channel attention map *M*_*c*_ ∈ *R* ^*C*×1×1^. Among them, *σ* is the sigmoid function.

Spatial feature extraction is as follows:

$$M_{s}\left(F\right)=\sigma \left(f^{7\times 7}\left(\left[AvgPool\left(F\right);MaxPool\left(F\right)\right]\right)\right),$$ (3)

Similar to the channel attention calculation, the average pooled features and the maximum pooled features are stitched together, and a 2D spatial attention map *M*_*s*_ ∈ *R* ^1×*H*×*W*^ is generated through the convolutional layer. Among them, *f* ^7 × 7^ represents a convolution operation with a convolution kernel size of 7*7.

In addition, for the local feature extraction module, the module spatially divides the input feature into four local features *F* and performs a two-layer convolution feature extraction module for each local feature *F*. Finally, we fuse the output features of the global attention module, the features of the local feature extraction module, and the output of the second residual block of the backbone network as the input of the subsequent residual module. The whole attention-based image feature extraction module fuses local and global features, so that the module not only pays attention to important global features but also does not miss local small features that may affect diagnosis.

##### 3.4.1.2 Blood testing feature extraction module

As a one-dimensional vector data, the blood detection indicator is prone to have the risk of over-fitting in the feature extraction process. To reasonably extract the features of blood test indicators, we use two layers of 1D convolution to extract one-dimensional vector data in the feature extraction module of blood test indicators, and there will be no overfitting problem.

##### 3.4.1.3 Modal fusion module

In the modality fusion part, we fuse the output of the attention-based image feature extraction module and the blood detection indicator extraction module, and input two fully connected layers to link the features to obtain the classification result. Specifically, the fully connected layer fuses the 12 features output from the attention-based image feature extraction module and the 3 features output from the blood detection indicator extraction module, and then extracts these features and obtains the category they belong to.

#### 3.4.2 Two-stage training strategy

**FIGURE 4:**
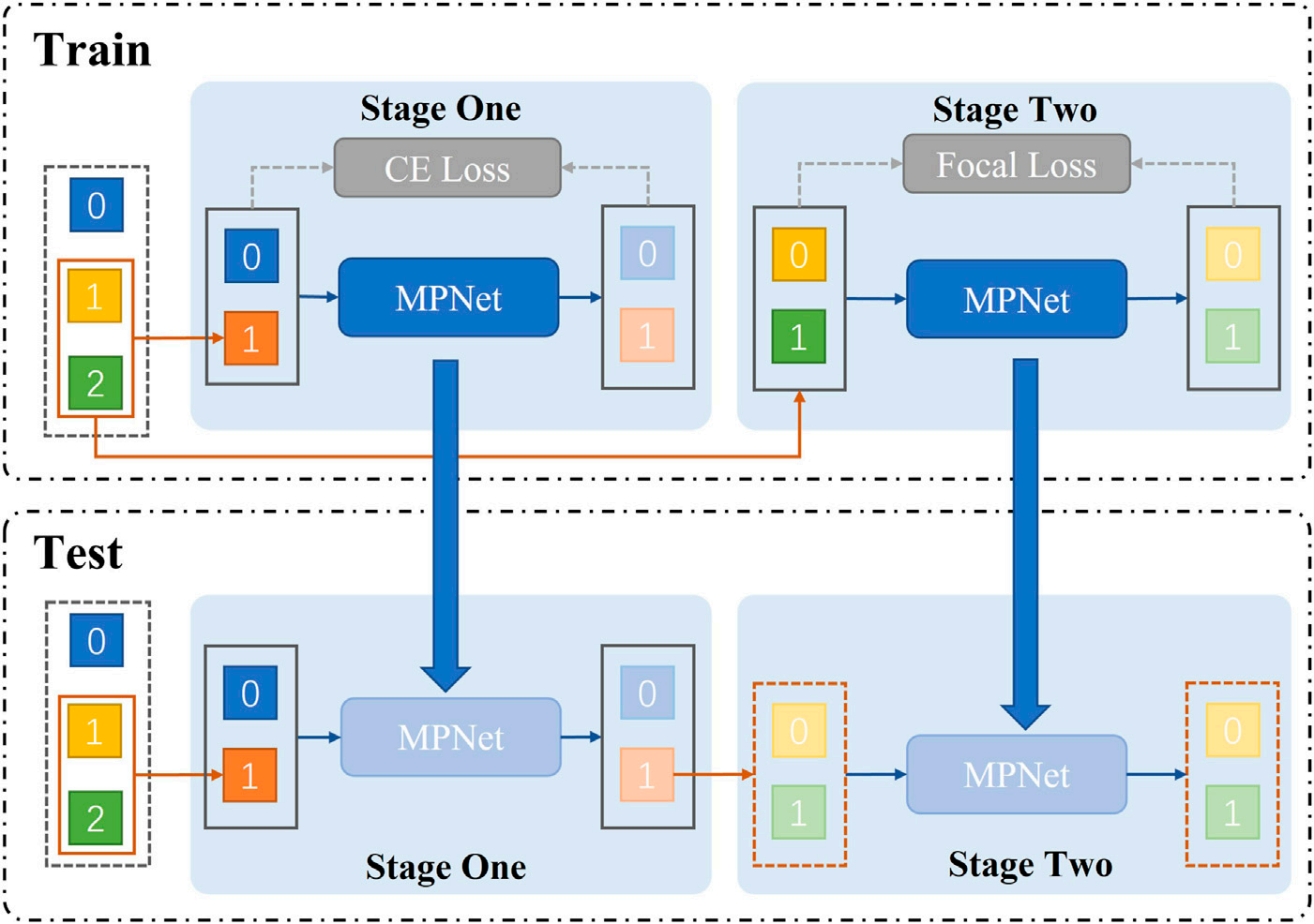
Train represents the two-stage structure of network training. First, the presence or absence of pneumonia is classified. Second, the type of pneumonia is classified. The test shows that we adopt a two-stage structure in the network test.

The model uses a two-stage joint training method to sequentially determine whether a patient has pneumonia. And it can further diagnose which type of pneumonia (bacterial pneumonia, viral pneumonia) the patient has (as shown in Figure 4). Specifically, during training, we use AMPNet to classify the raw labeled data once to distinguish normal samples from pneumonia samples. Then, the pneumonia samples from the first-stage classification are used as input to AMPNet to distinguish specific pneumonia types. In the first stage, we use the cross-entropy loss. In the second stage, we use focal loss (Lin et al., 2017) to classify the imbalanced harder samples. After a two-stage training strategy, the model obtains the final classification results (normal, bacterial pneumonia, and viral pneumonia). During testing, the models are tested in the same two-stage strategy. Furthermore, to further address the class imbalance problem of pneumonia samples, we use Mixup (Zhang et al., 2017) and Cutmix (Yun et al., 2019) data augmentation methods in two stages.

The details of the training are as follows: In the first stage, our learning rate is 0.05, and in the second stage, our learning rate is 0.005. The optimizer of both stages is SGD optimizer, and the training is 70 epochs. In addition, the model also adds Mixup, Cut, and Focal Loss, where the parameter of mixup is set to 0.06 and cutmix is set to 0.01, and focal loss is only used in the second stage with its gamma parameter set to 3, 0.25.

### 3.5 Computer hardware and software

All models are implemented using PyTorch and run on a server with 8 Nvidia GeForce 2080 GPUs. Each graphics card has 8192M of memory and the server has an Intel(R) Xeon(R) Silver 4110 CPU with 2.10GHz and 16G of RAM. In terms of software configuration, the CUDA version of the server is 10.2, and all codes are implemented in the Python language based on the PyTorch framework. The main Python libraries involved in the experiments are Numpy (for matrix operations), PIL (for reading, processing, and saving medical images), wandb (for tracking and analyzing experimental procedures), and torchvision (an image processing library related to PyTorch).

### 3.6 Evaluation indicators

To show the effectiveness of our models, we use the precision (Pre), recall (Rec), and F1 score (F1). Specifically, accuracy is a standard for measuring the percentage of correctly classified samples out of the total number of samples. Precision measures the percentage of true positive samples of all predicted positive samples. Recall has evaluated the probability that positive samples are correctly classified as positive. F1 score is the harmonic mean of Precision and Recall, which thus can evaluate the model’s performances more comprehensively from the perspectives of both Precision and Recall. The higher the values of these metrics are, the better the performance is. Formally.

$$Pre=\frac{TP}{TP+FP},$$ (4)

$$Rec=\frac{TP}{TP+FN},$$ (5)

$$F1\;score=\frac{Pre+Rec}{2*\left(Pre+Rec\right)}=\frac{2*TP}{T+P},$$ (6)

Where *TP*, *FP*, and *FN* are the number of true positive points, false positive points, and false negative points, respectively. *T* is the number of ground truth points of that class, and *P* is the number of predicted positive points. Due to the imbalanced data problem in the clinic pneumonia X-ray dataset, i.e., healthy data is more than pneumonia data, the general accuracy metric cannot adequately evaluate pneumonia classification performance. Furthermore, our goal is to achieve a three-class task that maximizes the true positive (TP) rate for pneumonia diagnosis. Therefore, we selected the F1-score, which balances recall and precision to evaluate TP, as our evaluation metric.

## 4 Results

### 4.1 Comparison of diagnostic performance of deep learning models under different datasets

As shown in Table 1, among the four commonly used convolutional neural network classification models, Seresnet50 has the best classification results in the Guangzhou Women and Children’s Medical Center Public Dataset (GZCMC). Average F1-score: 0.8420, Normal F1-score: 0.9488, Bacterial F1-score: 0.8660, Viral F1-score: 0.7111. There are good classification effects on all four models. However, when we performed the same classification experiments on a dataset collected by the Hainan Women’s and Children’s Medical Centre, which uses the pathogen-radiology-clinical diagnostic screening for definitive pneumonia classification, problems occurred. We found that the classification results decreased by at most 0.1933 in the average F1-score (on the VGG model). And the evaluation metric of each class decreased, especially in bacterial pneumonia and viral pneumonia (on the VGG model bacterial F1 -score decreased by 0.2396, and viral pneumonia even decreased by 0.3231). This suggests that although existing methods show good performance on public datasets, the performance is spurious, especially in distinguishing bacterial pneumonia from viral pneumonia. However, Seresnet50 has the highest F1-score for each class regardless of which dataset. This is one of the reasons why we propose that AMPNet choose it as the backbone network, the theoretical part of which has already been mentioned in the previous section. In conclusion, we find through rigorous experiments that the accuracy of the deep learning model in actual clinical practice is not high. This also shows that the actual disclosed method is difficult to apply in actual clinical practice. Therefore, in response to this problem, we carry out a study on the diagnosis of actual clinical pediatric pneumonia.

**TABLE 1:** Classification results of deep learning models under different datasets.

Class	Metric	VGG19		Densenet121		Resnet50		Seresnet50	
		Ourdataset	GZCMC	Ourdataset	GZCMC	Ourdataset	GZCMC	Ourdataset	GZCMC
Normal	Precision	0.9127	0.8974	0.9367	0.8987	0.9498	0.8912	0.9629	0.9342
	Recall	0.8989	0.9502	0.8882	0.9638	0.8896	0.9638	0.9055	0.9638
	F1-score	0.9057	0.9231	0.9118	0.9301	0.9187	0.9261	0.9333	0.9488
Bactieral	Precision	0.5891	0.8241	0.5814	0.8435	0.6279	0.8308	0.6434	0.8557
	Recall	0.6281	0.8725	0.6637	0.8947	0.6807	0.8947	0.7034	0.8765
	F1-score	0.6080	0.8476	0.6198	0.8684	0.6532	0.8616	0.6721	0.8660
Viral	Precision	0.3250	0.7070	0.3500	0.7820	0.2750	0.7711	0.3000	0.7395
	Recall	0.3171	0.5914	0.4516	0.6420	0.5789	0.6031	0.5455	0.6848
	F1-score	0.3210	0.6441	0.3944	0.7051	0.3729	0.6769	0.3871	0.7111
F1-score(avg)		0.6116	0.8049	0.6420	0.8354	0.6483	0.8215	0.6642	0.8420
D-value		0.1933		0.1934		0.1732		0.1778	

### 4.2 Comparison experiment of pneumonia diagnosis model based on X-ray image

To verify the capability of our proposed two-stage attention model for X-ray image feature extraction, we compared our model with the current SOTA models for pneumonia X-ray image diagnosis. Chouhan et al. (Chouhan et al., 2020) individually trained AlexNet, DenseNet121, Inception v3, GoogleNet50, and ResNet-18 on the training subset of the GWCMC dataset, and subsequently developed an ensemble model by majority voting. This model is called Multi-mode. CheXNet (Rajpurkar et al., 2017) is a method proposed by Rajpurkar P et al. based on the Densenet121 model. And it uses the Adam optimizer after ImageNet pre-training. It is a classic method for pneumonia diagnosis. (Vrbancic and Podgorelec, 2020). proposed to use a transfer learning-based approach by fine-tuning the medical dataset after pre-training with the ImageNet dataset. Then, Ayan et al. propose a convolutional neural network (CNN) ensemble method PNet (Ayan et al., 2022), which pre-trained on the ImageNet dataset were trained with the appropriate transfer learning and fine-tuning strategies on the chest X-ray dataset. To satisfy the requirements of the experiments, we choose to fine-tune the fully-connected layers on our dataset after pre-training on the GZCMC dataset.

**FIGURE 5:**
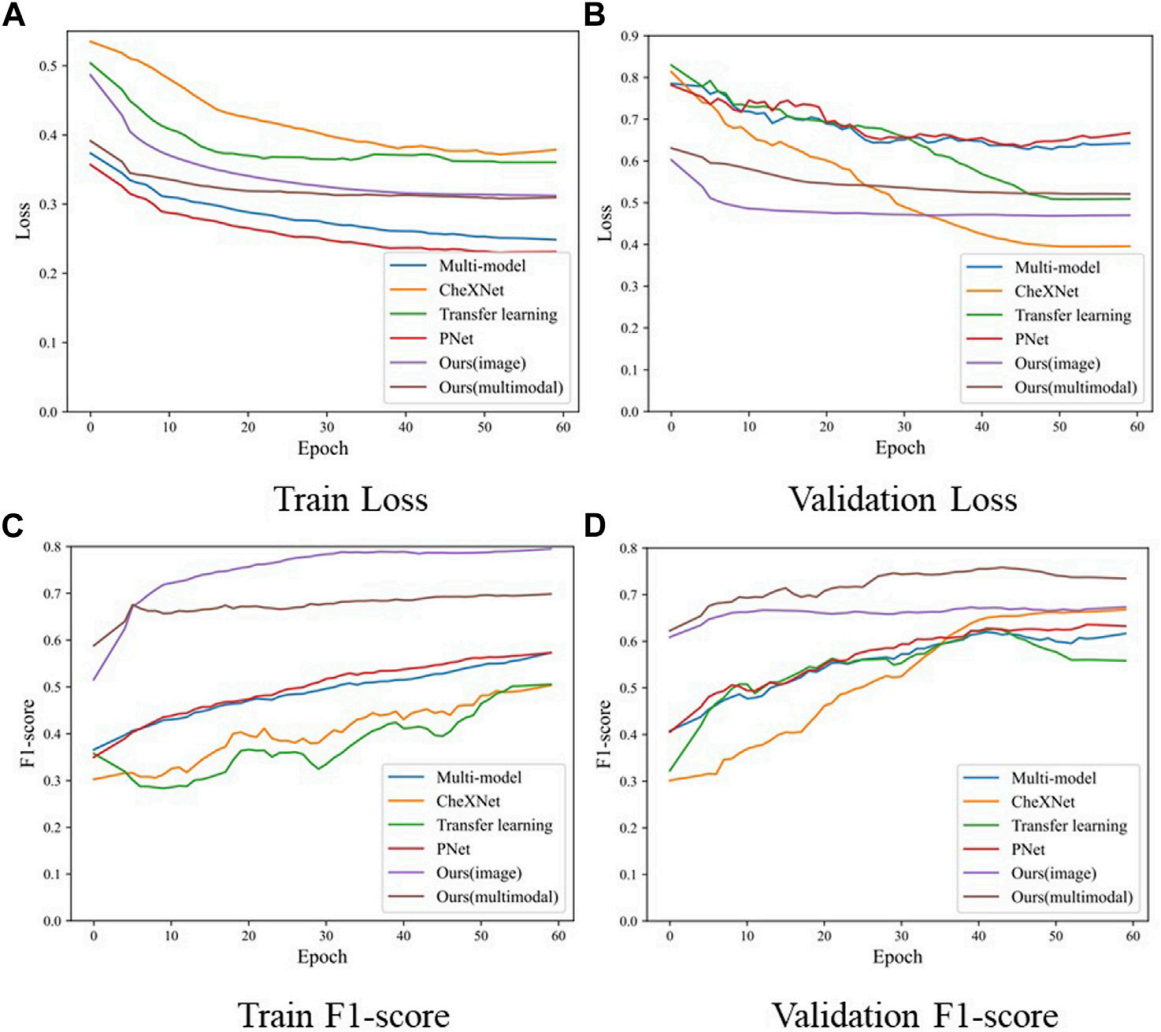
The loss and F1-score of the training process in the comparison experiment model, where **(A)** is the training loss, **(B)** is the validation loss, **(C)** is the F1-score of training samples, and **(D)** is the F1-score of validation samples.

The comparison experiment results are shown in the Table 2, and the training process of each model is shown in the Figure 5. During the training process, both the training loss and the verification loss gradually decrease and level off with each round. Although the verification loss fluctuates greatly for individual models in the initial stage, it still tends to be flat overall. The overall trend of training F1-score and verifying F1-score curves is also gradually increasing. However, in the verification F1-score curve, the transfer learning model shows a downward trend in the last 15 epochs, which may be the overfitting of the transfer learning model, resulting in a decline in the F1-score. In the comparative experiment results, the classification results of our model are improved by 1.86% compared with the SOTA model. On the one hand, the reason for this improvement is the proposed two-stage method reduces the difficulty of the model to directly perform three classification tasks. On the other hand, by distinguishing whether there is pneumonia or not at first, we reduce the missed diagnosis rate of pneumonia in patients. Our model improves the accuracy of the diagnosis of normal patients, which is 3.67% higher than the SOTA model. Furthermore, our model significantly improved the diagnosis of bacterial pneumonia (bacterial F1-score improved by 11.02% over the SOTA model). In general, our model outperforms the current SOTA model in overall diagnostic results for the lung X-ray imaging diagnosis task, which proves that our two-stage attention model for pneumonia diagnosis research is effective.

**TABLE 2:** Comparative experiment of X-ray diagnostic model of pediatric pneumonia, where the bold values (i.e., Ours (image)) are the best results using only single-modal data of images on our model, and Ours (multimodal) represents the best results by using multi-modal data of images and blood detection indicators on our model.

Method	F1-score(avg)	Normal			Bacterial			Viral		
		Precision	Recall	F1-score	Precision	Recall	F1-score	Precision	Recall	F1-score
Multi-model	0.6645	0.8865	0.9227	0.9042	0.7519	0.6063	0.6713	0.3500	0.5185	0.4179
CheXNet	0.6735	0.9074	0.98	0.9423	0.7317	0.6261	0.6748	0.5691	0.3125	0.4035
Transfer learning	0.6696	0.8981	0.9432	0.9201	0.7064	0.5969	0.6471	0.4594	0.4250	0.4416
PNet	0.6562	0.9258	0.8945	0.9099	0.5891	0.6609	0.6229	0.4250	0.4474	0.4359
Ours (image)	**0.6921**	**0.9960**	**0.9588**	**0.9790**	**0.7012**	**0.8915**	**0.7850**	**0.4167**	**0.2500**	**0.3125**
Ours (multimodal)	0.7781	0.9968	0.9956	0.9978	0.8438	0.8372	0.8405	0.4878	0.5000	0.4938

### 4.3 Ablation experiment of the two-stage multimodal pneumonia model

In this set of experiments, we first verified the effectiveness of our proposed multimodal model. As a result, we designed only images and blood detection vectors to compare two unimodal and multimodal models under a two-stage model. In addition, we also verify the effectiveness of our proposed modules based on multimodality, including the local attention module and the global attention module to improve feature extraction ability. Mixup and Cutmix hybrid data enhancement module and Focal loss module proposed for sample imbalance are also included.

In Table 3, the results in the multi-modal validation section show that multimodality has a significant improvement compared to single-modality. In terms of the average F1-score indicator, multimodality has an average improvement of 3.19% over blood detection vector modalities, and an increase of 4.03% compared with the imaging modality only. Especially in the diagnosis of viral patients, the multimodal model has a significant improvement. The multimodal viral F1-score is 17.03% higher than the blood detection vector modality and has an improvement of 13.9% compared to the image mode. The single modality can only distinguish whether there is pneumonia, but cannot distinguish whether it is viral or bacterial pneumonia. Even if the single modality has a good effect on bacterial diagnosis, it can be deceptive. The model is more likely to determine classification according to the party with the larger number of samples (i.e., bacterial pneumonia) because the samples are quite different and the information is incomplete. Therefore, we can conclude that multi-modal data brings more dimensional and rich information to the model than single-modal data. Therefore, the diagnostic model can integrate more data to improve the diagnostic accuracy of the pneumonia model. And it greatly improves the diagnostic performance for bacterial pneumonia and viral pneumonia, which are also difficult for doctors to distinguish.

**TABLE 3:** Ablation experiment of the two-stage multimodal pneumonia model, where bold values are the best results.

Class	Module	F1-socre(avg)	Normal			Bacterial			Viral		
			Precision	Recall	F1-score	Precision	Recall	F1-score	Precision	Recall	F1-score
Modal	image	0.6821	0.9998	0.9673	0.9834	0.7174	0.9496	0.8173	0.5000	0.1628	0.2456
	blood	0.6905	0.9899	0.9967	0.9923	0.7812	0.9495	0.8571	0.4615	0.1395	0.2143
	multi	0.7224	0.9807	0.9737	0.9766	0.7438	0.8561	0.7959	0.4285	0.3488	0.3846
Module	FL	0.7426	0.9998	0.9744	0.987	0.7692	0.6977	0.7317	0.4179	0.6512	0.5091
	Data Mix	0.7678	0.9998	0.9959	0.9979	0.8346	0.7986	0.8162	0.4509	0.5349	0.4894
	GLA	0.7597	0.9983	0.9836	0.9917	0.8014	0.7841	0.7927	0.4444	0.5581	0.4948
	FL + DataMix	0.7719	0.9998	0.9967	0.9989	0.8614	0.7692	0.8127	0.4028	0.6744	0.5043
	FL + GLA	0.7695	0.9999	0.9987	0.9993	0.8969	0.6793	0.7731	0.4125	0.7674	0.5366
	GLA + Data Mix	0.7694	0.9998	0.9930	0.9954	0.7746	0.8462	0.8088	0.4872	0.5219	0.5039
**ALL(ours)**		**0.7781**	**0.9968**	**0.9956**	**0.9978**	**0.8438**	**0.8372**	**0.8405**	**0.4878**	**0.5000**	**0.4938**

**FIGURE 6:**
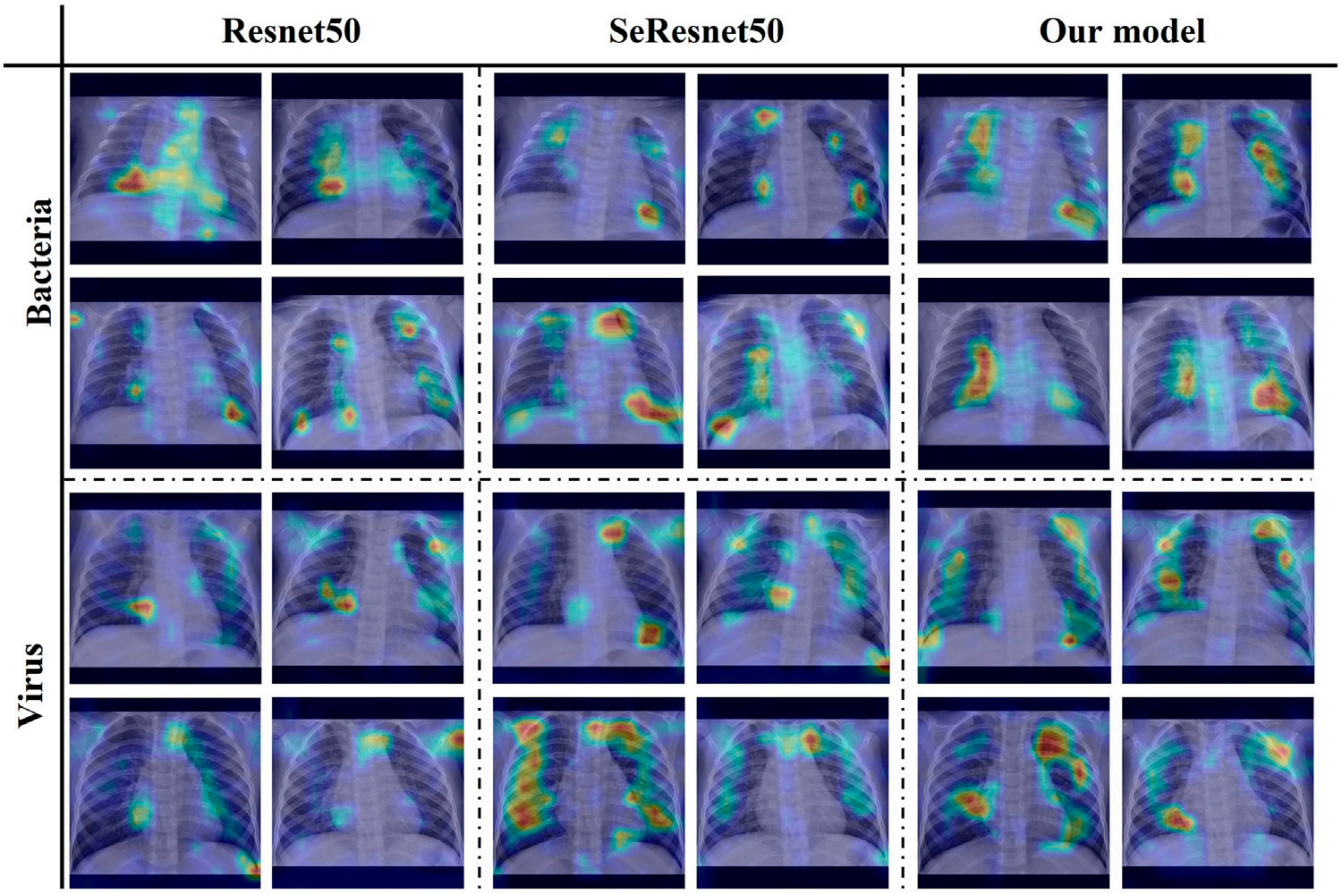
Attention map heatmap on three different models.

In addition, in the model module validation part of Table 3, the data of each module can show the effectiveness of module adding. And our proposed AMPNet has the best effect (average F1-score: 0.7781, normal F1-score: 0.9978, bacterial F1-score: 0.8405, viral F1-score: 0.4938). First, the data augmentation module of Mixup and Cutmix has the most significant overall improvement in model diagnosis. The average F1 score is improved by 4.54% relative to the base multimodal model. This shows that this data enhancement method can greatly improve the imbalanced data and X-ray clinical pneumonia data. Second, the local and global attention modules also have a good effect on the improvement of the overall performance of the model diagnosis. The average F1 score is improved by 3.73% relative to the base multimodal model. In addition, the performance of adding two modules is better than adding only one module, which also shows the effectiveness of three modules in improving the performance of the model. At the same time, it can be seen from the attention map of each model in Figure 6 that our proposed local overall attention module can learn features that are useful for model diagnosis more accurately. The attention map is no longer a piece of low-weight or high-weight but is distributed on both sides of the lung in a focused manner. This is also in line with the physician’s experience in X-ray reading of pneumonia.

### 4.4 Blind review of diagnostic results by radiologists

**FIGURE 7:**
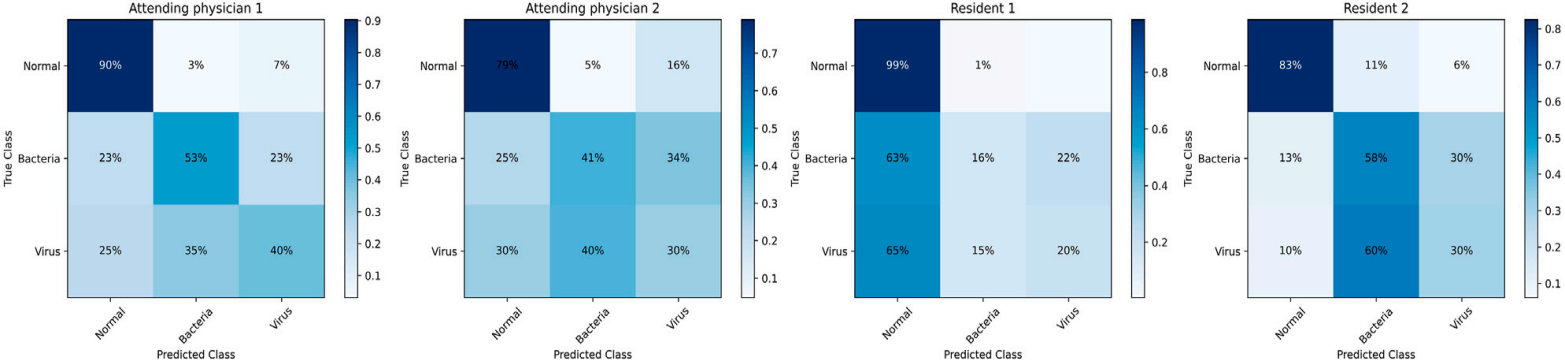
Confusion matrix of the diagnostic results of blind reviews by doctors with different seniority.

In this part, we make clinical diagnoses on the data of the Hainan Women’s and Children’s Medical Centre with the help of doctors. And we also explore the accuracy of doctors’ diagnoses based on the Hainan Women’s and Children’s Medical Centre dataset with other clinical diagnosis information. Four radiologists with different seniority reviewed the patient’s X-ray images and blood tests to make diagnoses. Due to limited physician review efforts, we randomly selected half of the patient data in the testing set. Two of the doctors are radiology residents (Residents 1 and 2 have 3 and 4 years of chest image interpretation experience respectively). The other two are attending radiologists (8 and 7 years of chest image interpretation experience for Attending 1 and 2 respectively). They are all blinded to clinical data and previous imaging findings. The results of the blind review are shown in Table 4, among which the attending doctor 1 has the best diagnosis results. (F1-score (avg): 0.5970, normal: precision 0.9039, recall 0.9119, F1- score 0.9079, bacterial: precision 0.5312, recall 0.7083, F1-score 0.6071, virality: precision 0.4000, recall 0.2105, F1-score 0.2759). Figure 7 shows the confusion matrix of the results of each physician’s review. According to the diagnosis results of the three groups, physicians have a considerable ability to diagnose whether the patient has pneumonia. But when physicians face the diagnosis of bacterial pneumonia and viral pneumonia, the results are more dependent on seniority experience. Specifically, the doctor with the longest experience is better at distinguishing bacteria from pneumonia (average F1-score of the four physicians, attending 1:0.5970, attending 2:0.4854, resident 1:0.4494, resident 2:0.5396). According to the diagnosis results of all physicians in the chart, it can be concluded that physicians with relatively high seniority have higher overall accuracy, which is in line with the physician’s review rules. However, the best results which doctors came to by reviewing imaging and blood test data are still not as accurate as those obtained by deep learning models from Guangzhou Women’s and Children’s Medical Center (GZCMC) and Hainan Women’s and Children’s Medical Centre data. Therefore, it is meaningful for us to study a pneumonia diagnosis model with better performance.

**TABLE 4:** Diagnostic results of blind reviews by doctors with different seniority.

Method	F1-score(avg)	Normal			Bacterial			Viral		
		Precision	Recall	F1-score	Precision	Recall	F1-score	Precision	Recall	F1-score
Attending 1	0.5970	0.9039	0.9119	0.9079	0.5312	0.7083	0.6071	0.4000	0.2105	0.2759
Attending 2	0.4854	0.7904	0.8916	0.8380	0.4063	0.5778	0.4771	0.3000	0.0923	0.1412
Resident 1	0.4494	0.9869	0.8100	0.8898	0.1563	0.6667	0.2532	0.2000	0.2105	0.2051
Resident 2	0.5396	0.8253	0.9497	0.8832	0.5781	0.4933	0.5324	0.3000	0.1538	0.2034

### 4.5 Pneumonia Diagnostic Model Experiment Guides Clinical Practices.

In order to explore the influence of various physiological indicators of blood detection on the pneumonia diagnosis model, whether various detection indicators can provide more feasible suggestions for doctors’ clinical diagnosis under the results of the deep learning model. We eliminated the factor of age and performed four groups of ablation experiments for the remaining indicators, namely, without white blood cells (Wo WBC), without neutrophils (Wo Neut), without C-reactive protein (Wo CRP), without procalcitonin (Wo PCT).

Based on the analysis of the experimental results in Table 5, we found that the single absence of each of the four blood examination indicators showed different degrees of decrease in the accuracy of the diagnostic results. It confirms that the combined analysis of WBC, Neut, CRP, and PCT is significantly effective in improving the accuracy of pneumonia diagnosis in medicine. However, it is medically impossible to state the extent to which each indicator affects the diagnostic result. In the results of the ablation experiment in Table 5, we can see that the Neut indicator is the most significant decrease in the overall classification effect in the task of identifying healthy, bacterial pneumonia, and viral pneumonia (5.58% overall decrease in the F1-score (avg)). It indicates that Neut as an important component of total leukocyte count is more effective in diagnosing the three categories of healthy, bacterial, and viral pneumonia relative to the other three indicators. According to the experimental results, the Wo WBC indicator has a greater impact on the diagnosis of bacterial pneumonia than the Wo Neut indicator, which is supported by having the minimum *p*-value between their missing experiments as 0.2962, proving that the WBC indicator has a greater value in bacterial diagnosis. In addition, the minimum *p*-value between the results of the Wo Neut and Wo CPR is 0.1781, proving that the Wo Neut indicator have a greater impact on the diagnostic effect of viral pneumonia than the Wo CPR indicators. Please do note that although the *p*-values between the results four blood-related indicators are generally larger than the “gold standard” (i.e., *p*-values are generally higher 0.05), the *p*-values are small enough to show the different influence (even if the differences may not be statistically significant) of these four blood-related indicators on pneumonia diagnosis.

**TABLE 5:** Diagnostic results of blind reviews by doctors with different seniority, where bold values are the best results.

Indicators	F1-score(avg)	Normal			Bacterial			Viral		
		Precision	Recall	F1-score	Precision	Recall	F1-score	Precision	Recall	F1-score
Wo WBC	0.7222	0.9979	0.9940	0.9956	0.8198	0.7054	0.7583	0.3448	0.5000	0.4082
Wo Neut	0.7274	0.9892	0.9926	0.9909	0.8151	0.7519	0.7823	0.3600	0.4500	0.3999
Wo CRP	0.7397	0.9986	0.9978	0.9989	0.8349	0.7054	0.7647	0.3770	0.5750	0.4554
Wo PCT	0.7361	0.9984	0.9978	0.9989	0.8381	0.6822	0.7521	0.3692	0.6000	0.4571
Ours	**0.7781**	**0.9968**	**0.9956**	**0.9978**	**0.8438**	**0.8372**	**0.8405**	**0.4878**	**0.5000**	**0.4938**

## 5 Concusion and future work

In our study, by comparing the pathogen-radiology-clinical diagnostic screening dataset with existing public datasets under commonly used deep learning models, we identified the problem that current public data are unreliable. To investigate more efficient and accurate models based on existing clinical data, we propose has two-stage attention multimodal pneumonia classification model. Then, our model achieves state-of-the-art results on the task of diagnosing pneumonia on lung X-ray images, with an average F1-score improvement of 2.76% compared to existing SOTA work. At the same time, we found that the two-stage strategy can reduce the misdiagnosis rate of the pneumonia model. Our proposed two-stage model is effective for difficult clinical datasets. Then, we demonstrate the effectiveness of each module of the AMPNet model through ablation experiments. Overall, our study provides a plausible explanation for the dataset study and for the first time proposes an excellent multimodal pneumonia diagnosis model. At the same time, the model outperformed the blind review results of radiologists by a wide margin. Another contribution of our study is on experiments and statistical analysis of the impact of blood test indicators on classification results. Further, we propose possible recommendations that could provide guidance for professional radiologists in the diagnosis of pneumonia, especially bacterial and viral pneumonia. These recommendations provide a feasible direction for future research on pneumonia diagnosis.

Our study provides a more efficient model and some promising recommendations for physicians currently diagnosing pediatric pneumonia. However, we have not thought of a better solution for the current extremely imbalanced pneumonia data. Especially when bacterial pneumonia and viral pneumonia are indistinguishable, the data gap between bacterial pneumonia and viral pneumonia is too large. This situation is very common in clinical, and it is also a problem that we need to further solve.

## Data Availability

Publicly available datasets were analyzed in this study. This data can be found here: https://www.kaggle.com/datasets/kostasdiamantaras/chest-xrays-bacterial-viral-pneumonia-normal.
